# Cat-Scratch Disease

**Published:** 1954-10

**Authors:** D. Bryant, R. P. Warin

**Affiliations:** Department of Dermatology, United Bristol Hospitals; Department of Dermatology, United Bristol Hospitals


### CAT-SCRATCH DISEASE

### (La maladie des griffes du chat)

BY

D. BRYANT, M.B., B.CHIR.

and

R. P. WARIN, M.D., M.R.C.P.

(*Department of Dermatology, United Bristol Hospitals*)

The purpose of this article is to draw attention to a disease which has only recently been recognized in this country. The main features of the condition are described and a probable case arising in the Bristol area is reported.

In 1947, during conversations between Debre of Paris and Foshay of Cincinnati, it became apparent that both these workers had seen numerous examples of this condition during the preceding twenty years (Daniels and MacMurray, 1951). Once its existence was recognized case reports began to appear in the literature, the majority from France and the United States, but also from India (Debre *et al.*, 1950a, 1950b; Mollaret *et al.*, 1950; Daniels and MacMurray, 1951; and Greer and Keefer, 1951. In 1952 an annotation appeared in *The Lancet* and following this cases have been reported in this country (Cox, 1952; Campbell and Wheatson, 1952; and Bettley and Fairburn, 1953).

Daniels and MacMurray (1951) give a full account of the clinical findings in this condition. Many of the cases follow a few days after the scratch or bite of a cat, but there are a large number reported without such a history. At the site of the scratch about half the cases develop an erythematous plaque or nodule on which a vesicle or pustule may appear. Some four to twenty-eight days after the injury a regional adenitis occurs. Commonly one or two lymph nodes are affected but adjacent groups may also become involved, particularly in the neck. The swelling is sometimes quite large (a tangerine and a golf ball are mentioned in the literature) and may become red and tender. Suppuration may occur. The lymphadenopathy slowly settles down over a period of weeks or months.

The patients do not appear to be severely ill, but about half have some fever at the onset. Headaches and malaise are often present. In a few cases a widespread eruption may appear early in the illness. This may be macular or vesicular but erythema multiforme (Greer and Keefer, 1951) erythema nodosum (Cox, 1952) and a papular urticaria-like eruption (Bettley and Fairburn, 1953) have been described.

The blood leucocyte count may show a slight increase in the acute phase but there is no constant abnormality present. Pus, if present, reveals no bacteria and cultures are negative. There is a well-marked reaction to an intradermal injection of an antigen prepared from pus obtained from a lymph node. This takes the form of an indurated papule appearing within forty-eight hours and lasting six to eight weeks. Bettley and Fairburn (1953) have tested the antigen on forty normal adults; three of these showed a positive response but in each case a history of close contact with cats was obtained. Willcox (1952) obtained cross positives in non-specific urethritis but apart from this it would appear that a positive intradermal reaction is good evidence of the disease although of course it would presumably also indicate past infection. The microscopic appearance of the lymph node is not diagnostic but follows a certain pattern. In mild cases nodules of reticular cell hyperplasia are present. In more advanced cases the centres of the nodules have become necrotic and neutrophil leucocytes mingle with pyknotic debris. The centres of necrosis coalesce into an eosinophilic mass around the borders of which there may be epitheloid cells and giant cells.

A number of conditions have to be considered in the differential diagnosis. The disease is likely to run a more prolonged and less active course than acute bacterial lymphadenitis and there is commonly no neutrophil leucocytosis. On the other hand tuberculosis as a primary focus or as scrofula is a more persistent condition. A tuberculin skin test will be of considerable help (particularly in children). In localities where tularemia occurs this condition may be excluded by agglutination reactions. Lymphogranuloma inguinale may have to be considered and the Frei test is helpful in distinguishing it.

There is no specific treatment. Aureomycin is reported to have been successful in some cases (Debre *et al.*, 1950a) but, in a self-limited condition, assessment of the value of aureomycin must be very difficult. Other antibiotics and sulphonamides are reported as being with effect. The aetiology of the disease is unknown but its general behaviour does suggest a virus infection perhaps of the lymphogranuloma-psittacosis group. Mollaret was able to transmit the disease to one human volunteer out of four, but had more success with apes (Mollaret *et al.*, 1951a and 1951b). Transmission to cats was unsuccessful which suggests that the cat is only a vector.

It is difficult to assess the incidence of the condition. In the past many cases must have been misdiagnosed and it is felt that now that the condition is recognized a number of cases will come to light. Bettley and Fairburn's work would suggest that some 7–8 per cent of adults have had the disease and it may well prove to be a very common benign infection. It is possible that the incidence of the condition has increased in the last few years and this may be due to a habit of closer contact of man with his household pets. It is with the object of drawing attention to the possibility of this infection arising in the West Country that this case report is recorded.

### CASE REPORT

A girl of five was brought to the Out-Patient Department with a complaint that there was a spot on the right side of her neck with some enlargement of the glands in the neck. Two to three weeks before the mother had noticed a small pimple on the right side of the child's neck. This had persisted but had not formed a pustule or ulcerated. The child was quite well at the time and her temperature had not been taken.

There was no family history of tuberculosis. The family possessed a male kitten of ten months with which the child played.

On examination there was a small area of erythema, with some induration, behind the right sternomastoid muscle and about two inches above the clavicle. There was enlargement of the right supraclavicular lymph node. This measured about 3 cm. by 2 cm. and was not tender. It was attached deeply but was not fixed to the skin. There was also enlargement of the left submandibular lymph node which showed similar characteristics. No other lymph nodes were enlarged. The spleen and liver were not palpable. The blood leucocyte count was 10,000 (neutrophil 60 per cent). A tuberculin jelly test was negative.

Antigen, specific for cat-scratch disease, was obtained from Dr. Ray Bettley. Intradermal injection of 0·1 ml. of this was followed within twenty-four hours by an area of erythema at the site of injection, which increased to 3 cm. in diameter. One week after the injection a firm, intradermal papule, measuring 1 cm. was palpable. There was no local adenitis. Three weeks after the injection, this papule, though much smaller, was still palpable.

A month after the child's first visit there was little change in the scar on the neck, but the two enlarged lymph nodes were much smaller and were no longer fixed deeply.

SUMMARY

(1) Cat-scratch disease has been recognized as a clinical entity in the past eight years.
(2) The main features of the condition are described.
(3) It is probable that the condition is quite common but not diagnosed as such.
(4) A case arising in the Bristol area is reported.

REFERENCES

Bettley, F., and Fairburn, E. A. (1953). *Lancet*, **1**, 520.

Campbell, E. S. K., and Wheaton, C. E. (1952). *Lancet*, **1**, 975.

Cox, P. J. N. (1952). *Proc. Royl. Soc. Med.* **45**, 456.

Daniels, W. B., and MacMurray, F. G. (1951). *Arch. Int. Med.*, **88**, 736.

Debre, R., Lamy, M., Jammet, M. L., Costil, L., and Mozziconacci, P. (1950a). *Bull. Soc. med. Hôp. Paris*, **66**, 76 (1950a).

Debre *et al.* (1950b). *Semaine Hôp. Paris*, **26**, 1895.

Greer, W. E. R., and Keefer, C. S. (1951). *New Eng. J. Med.*, **244**, 545.

Mollaret, P., Reilly, J., Bastin, R., Tournier, P. *Bull. Soc. med. Hôp. Paris*, **66**, 424 (1950).

Mollaret *et al.* (1951a). *Presse med.*, **59**, 681.

Mollaret *et al.* (1951b). Ibid, **59**, 701.

Willcox, R. R. (1952). *Lancet*, **1**, 470.

